# Inhibiting efferocytosis reverses macrophage-mediated immunosuppression in the leukemia microenvironment

**DOI:** 10.3389/fimmu.2023.1146721

**Published:** 2023-03-07

**Authors:** Joselyn Cruz Cruz, Kristen C. Allison, Lauren S. Page, Alexis J. Jenkins, Xiaodong Wang, H. Shelton Earp, Stephen V. Frye, Douglas K. Graham, Michael R. Verneris, Alisa B. Lee-Sherick

**Affiliations:** ^1^ Division of Pediatric Hematology, Oncology, and Bone Marrow Transplant, University of Colorado, Aurora, CO, United States; ^2^ Center for Integrative Chemical Biology and Drug Discovery, University of North Carolina at Chapel Hill, Chapel Hill, NC, United States; ^3^ Lineberger Comprehensive Cancer Center, Departments of Medicine and Pharmacology, University of North Carolina at Chapel Hill, Chapel Hill, NC, United States; ^4^ Department of Pediatrics, Emory University, Atlanta, GA, United States

**Keywords:** MERTK, Mertk inhibitors, tumor-associated macrophages, efferocytosis, acute myeloid leukemia, leukemia associated macrophages, leukemia microenvironment, tumor micro environment (TME)

## Abstract

**Background:**

Previous studies show that the spleen and bone marrow can serve as leukemia microenvironments in which macrophages play a significant role in immune evasion and chemoresistance. We hypothesized that the macrophage driven tolerogenic process of efferocytosis is a major contributor to the immunosuppressive leukemia microenvironment and that this was driven by aberrant phosphatidylserine expression from cell turnover and cell membrane dysregulation.

**Methods:**

Since MerTK is the prototypic efferocytosis receptor, we assessed whether the MerTK inhibitor MRX2843, which is currently in clinical trials, would reverse immune evasion and enhance immune-mediated clearance of leukemia cells.

**Results:**

We found that inhibition of MerTK decreased leukemia-associated macrophage expression of M2 markers PD-L1, PD-L2, Tim-3, CD163 and Arginase-1 compared to vehicle-treated controls. Additionally, MerTK inhibition led to M1 macrophage repolarization including elevated CD86 and HLA-DR expression, and increased production of T cell activating cytokines, including IFN-β, IL-18, and IL-1β through activation of NF-κB. Collectively, this macrophage repolarization had downstream effects on T cells within the leukemia microenvironment, including decreased PD-1^+^Tim-3^+^ and LAG3^+^ checkpoint expression, and increased CD69^+^CD107a^+^ expression.

**Discussion:**

These results demonstrate that MerTK inhibition using MRX2843 altered the leukemia microenvironment from tumor-permissive toward immune responsiveness to leukemia and culminated in improved immune-mediated clearance of AML.

## Introduction

Efferocytosis is a tolerogenic process where macrophages phagocytose apoptotic cells, mediated by the binding of phosphatidylserine (Ptdser) on apoptotic cells to MerTK (or other efferocytic receptors) on macrophages *via* bridging molecules such as Gas6 and Pros1. Efferocytosis inhibits T cell activation and limits tissue damage in the resolution of inflammation/injury by removing self-antigen and secretion of T cell suppressive cytokines. However, Ptdser surface expression – which is usually tightly regulated (i.e., retained on the inner plasma membrane) in healthy cells – becomes externalized during apoptosis or with rapid cell turnover, such as in tumorigenesis (1). In fact, several studies have demonstrated that viable non-apoptotic cancer cells express high levels of PtdSer due to dysregulation of calcium-dependent flippase activity (2–4). Specifically, high levels of PtdSer are often expressed on acute leukemia cells (4, 5).

It has been proposed that efferocytosis in the solid tumor microenvironment skews tumor-associated macrophages (TAMs) toward a M2-like polarization and subverts T cell responses, aiding in tumor growth (6–9) and metastatic spread (10). Tumor-permissive macrophages have been identified within solid tumors and more recently in the spleen and bone marrow (BM) – creating a leukemia tumor microenvironment (11, 12). These leukemia-associated macrophages (LAMs) are phenotypically similar to wound healing (M2) macrophages and have been implicated in immune evasion, chemotherapy resistance, and extramedullary spread of leukemia (12–15). Due to the basal overexpression of PtdSer on leukemia cells, in addition to the significant cell turnover caused by rapid and ineffective hematopoiesis of leukemic blasts, the leukemia microenvironment contains abundant efferocytosis signals. Given that LAMs express the prototypic efferocytic receptor MerTK, we sought to evaluate whether targeting MerTK-dependent efferocytosis by LAMs would diminish leukemia growth through skewing of the leukemia microenvironment using a novel clinical grade small molecule tyrosine kinase, MRX2843.

Previously, we identified MerTK as a cell intrinsic therapeutic target, given that its expression on leukemia cells confers a survival advantage and chemoresistance *in vitro* and *in vivo (* 16–19 *).* These discoveries led to the development of type 1 MerTK small molecule inhibitors, including MRX2843, which is currently being tested in early phase clinical trials (NCT03510104, NCT04872478, NCT04762199) for its inhibitory effects on MerTK and FLT3. Using MRX2843 to block efferocytosis in syngeneic murine models of acute myeloid leukemia (AML) and using human samples *in vitro*, we demonstrate that MRX2843 skews M2-like macrophages toward M1 polarization. The resultant altered leukemia microenvironment led to activation of T cells, which lack MerTK. The combined effect on LAMs and the surrounding leukemia microenvironment led to AML clearance *in vivo*. These results were independent of MerTK inhibition on AML cells, given that the AML cell lines used in these experiments expressed little to no MerTK. Thus, targeting LAM-associated MerTK, using MRX2843 could have a novel use: repolarizing the leukemia microenvironment through blocking efferocytosis.

## Methods

### Cell culture

Murine AML cell line harboring a t(11;19) *KMT2A-MLLT1* translocation (“MLL-ENL”), and primary murine AML harboring a t(11;19) *KMT2A-MLLT3* translocation (“MLL-AF9”) were gifts from Deb DeRyckere. C1498 and Kasumi-1 were purchased from ATCC. Kasumi-1 cell identity was confirmed using short tandem repeat microsatellite loci analysis. Cell lines were maintained in RPMI medium plus 10% FBS and penicillin/streptomycin (cRPMI). MLL-AF9 cells were thawed and used immediately; they did not survive *in vitro* culture. For *in vitro* studies, MRX2843 was dissolved in dimethyl sulfoxide (DMSO; Sigma); vehicle controls were administered the equivalent volume of DMSO.

### Murine AML models

C57BL/6J, *Mertk*-null (B6;129-*Mertk^tm1Grl^*/J), and TCRα^-/-^ (B6.129S2-Tcra^tm1Mom^/J) mice were purchased from Jackson Laboratory and bred in-house. *Mertk*-null mice were backcrossed with C57BL/6J mice for ≥12 generations (*Mertk^–/–^*). *Mertk* genotype was verified prior to use; control mice were MerTK-wildtype littermates (*Mertk^WT^)* with identical backcrossing. LysM-Cre (*Lyz2^tm1(cre/ERT2)Grtn^*/J) mice were obtained from Jackson Laboratory and crossed with MerTK-loxp mice (gift from Carla Rothlin); after genotype verification, mice were treated with tamoxifen as previously described (20). Mice were inoculated with AML cells *via* tail vein at 6-8 weeks of life. For *in vivo* studies, MRX2843 was dissolved in phosphate buffered saline (PBS). Beginning three days after leukemia inoculation, MRX2843 (60mg/kg) or PBS was administered daily *via* oral gavage. Once mice developed advanced leukemia (>20% weight loss, hind-limb paralysis, inactivity) they were euthanized, and duration of survival was recorded. Alternatively, three weeks after starting MRX2843, mice were euthanized; peripheral blood, BM and spleen were harvested for analysis. Animal experiments were conducted in accordance with University of Colorado IACUC regulatory standards.

### Macrophage and AML co-cultures

Bone marrow derived macrophages (BMDM) were derived from isolated monocytes from the marrow of C57BL/6 mice using EasySep Mouse CD11b Positive Selection Kit II (Stemcell Technologies). Alternatively, human monocytes were isolated from discarded de-identified blood donation leukopaks from the Children’s Hospital Colorado Blood Donor Center, after written informed consent in accordance with the Declaration of Helsinki. Leukopak peripheral blood mononuclear cells (PBMCs), obtained using Lymphoprep (Stemcell Technologies), underwent CD14^+^ isolation using MojoSort™ Human CD14 selection kit (Biolegend). Monocytes were matured to macrophages on non-TC treated plates for three to seven days in cRPMI supplemented with 25ng/mL GM-CSF and 5ng/mL M-CSF, after which non-adherent cells were discarded. PtdSer expression on AML cell lines was induced *via* UV light exposure (15 minutes), followed by incubation at 37° for two to four hours, and then opsonized with 250nM recombinant mGAS6 (R&D Systems). Macrophages were pretreated with 300nM MRX2843 or vehicle for one hour in 24 well plates before AML cells were added (1.0×10^6^ cells/well). After 48 hours, non-adherent AML cells in the supernatant were removed; macrophages were washed with PBS thrice to remove residual AML cells.

### Mixed leukocyte reaction

Flat-bottom 96-well plates (Corning) were coated with 10 µg/mL anti-CD3e (clone OKT3; Thermo Fisher Scientific) in sterile PBS overnight at 4°C and then washed to remove unbound antibody. T cells were isolated from PBMCs using EasySep™ Human T Cell negative selection kit (Stemcell Technologies) and then cultured on the anti-CD3e coated plates (2x10^5^ cells/well) in cRPMI media. Primed T cells were collected after 48 hours and added to macrophage/leukemia co-cultures in equal proportions (1:1:1 ratio) for 48 hours at 37˚C.

### Flow cytometry

Harvested cells were treated with Fixation/Permeabilization Kit reagent (BD Biosciences). Immune cell characterization was quantitated on a Gallios (Beckman Coulter) flow cytometer and analyzed using Kaluza software, or a Cytec Aurora (Cytec) spectral flow cytometer and analyzed using FCS Express software (DeNovo Software). Antibodies listed in **Supplemental Table 1** were used according to manufacturer recommendations.

### Immunoblot analysis

Whole cell lysates were prepared after two to six hours of co-culture, and proteins were resolved as previously described (18), probed with antibodies in **Supplemental Table 1** according to manufacturer recommendations and visualized by horseradish peroxidase chemiluminescence (Perkin-Elmer).

### Real-time quantitative RT-PCR

After 12 hours of macrophage and AML cell co-culture, RNA was isolated from harvested macrophages using RNeasy Plus Mini Kit (Qiagen). Real-time reverse-transcription polymerase chain reaction (RT-PCR) analysis was performed using TaqMan Universal PCR Master Mix with primers in **Supplemental Table 1**. Threshold cycle values were normalized to the GAPDH RNA internal control, and analysis was performed by comparing MRX2843 to vehicle values.

### Cytokine analysis

Peripheral serum collected from *in vivo* assays was analyzed for cytokine concentrations with two independent ProcartaPlex immunoassays using the Luminex MAGPIX Instrument System (Luminex).

### Statistics

Statistical analyses were performed using GraphPad Prism software (v6.05). One-way ANOVA corrected with Bonferroni’s multiple comparisons test was used for experiments with three treatment groups. Student’s t-test was used when two samples were analyzed. Results indicate the mean values and were considered significant when p<0.05.

## Results

### Inhibition of MerTK in leukemia-associated macrophages improves survival in leukemic mice

**Figure 1:**
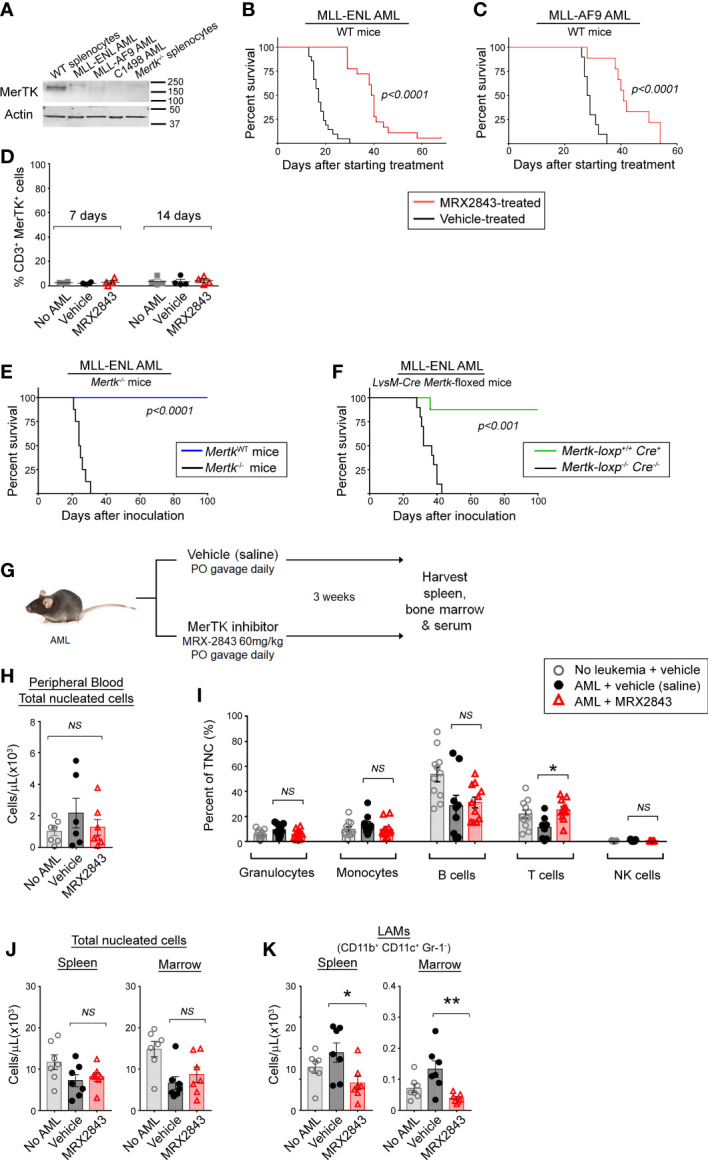
Inhibition of MerTK on leukemia-associated macrophages increases survival in syngeneic murine models of AML. **(A)** MerTK expression in murine AML cell lines was determined by immunoblot. Splenocytes from a *Mertk* wild-type (WT splenocytes) and *Mertk*-null (*Mertk^-/-^* splenocytes) mouse were used as positive and negative controls, respectively. Actin is shown as a loading control. **(B, C)** Syngeneic AML cells were inoculated into C57BL/6 mice by tail vein injection. After three days, mice began daily treatment with MerTK inhibitor MRX2843 (60mg/kg; red line) or an equivalent volume of vehicle (saline; black line). **(B)** Mice inoculated with 1x10^5^ MLL-ENL cells (vehicle n =21, MRX2843 n =18; three independent replicates). **(C)** Mice inoculated with 1x10^5^ MLL-AF9 cells (vehicle n = 10, MRX2943 n = 9; two independent replicates). **(D)** Peripheral blood T cells collected from mice 7 days and 14 days after starting treatment was analyzed by flow cytometry for MerTK expression on CD3^+^ T cells. **(E)** 1x10^5^ MLL-ENL AML cells were inoculated into *Mertk^-/-^* mice (blue line) or littermate wild-type controls (*MerTK^WT^*; black line) by tail vein injection (*MerTK^WT^* n =7, *Mertk^-/-^* n =8; two independent replicates). **(F)** MLL-ENL AML cells were inoculated into mice by tail vein injection after genotype confirmation of MerTK knock-down in dual MerTK and LysM expressing macrophages (i.e. *Mertk-loxp* ^+/+^ *Cre* ^+/- or +/+^, n = 19). Control littermate mice (*Mertk-loxp* ^-/-^ *Cre* ^-/-^, n = 19) did not have MerTK knockdown (two independent replicates). **(B–F)** Weight loss and health score were monitored as surrogates for disease burden; Kaplan-Meier survival analysis measured leukemia-free survival. Analyzed using log-rank test, comparing MerTK inhibited (MRX2843, *Mertk^-/-^, Mertk-loxp* ^+/+^ *Cre* ^+^) to controls (vehicle, *MerTK^WT^, Mertk-loxp* ^-/-^ *Cre* ^-/-^). **(G)** Diagram of the murine model used for flow cytometric evaluation in this manuscript. **(H-K)** C57BL/6 mice inoculated with MLL-ENL AML and treated daily with MerTK inhibitor MRX2843 (red triangles) or vehicle (saline; black circles); mice not inoculated with leukemia were used as a control (open gray circles). **(H)** Peripheral blood total viable nucleated white blood cell count was quantified by trypan blue exclusion, and **(I)** percentages of granulocytes, monocytes, B cells, T cells, and NK cells were quantified by flow cytometric analysis. **(J)** Total viable nucleated cell counts of the spleen and bone marrow were quantified by trypan blue exclusion. **(K)** Harvested cells were assessed by flow cytometric analysis, and leukemia-associated macrophages (LAMs) within the spleen and marrow were quantified. Analyzed using 1-way ANOVA. (*p<0.05, **p<0.01, NS, not significant).

To evaluate MerTK inhibition on leukemia-associated macrophages (LAMs) *in vivo* without the confounding factor of cell intrinsic effects, we utilized murine AML cell lines and primary murine AML which had very little or no MerTK expression compared to splenic macrophages (**Figure 1A**).

Leukemia was allowed to establish in syngeneic immunocompetent C57BL/6 mice for three days, and then treatment with MRX2843, a highly specific small molecule MerTK tyrosine kinase inhibitor (MerTKI), was initiated *via* daily oral gavage at 60mg/kg – a dose that is known to inhibit MerTK *in vivo*, and has minimal toxic effects (21). Mice were monitored daily and euthanized once they developed symptoms of leukemia. In mice inoculated with MLL-ENL cells, treatment with the MerTKI was associated with a significant median survival prolongation compared to vehicle (40 vs 17 days, p<0.0001) (**Figure 1B**). These results were validated in identical survival studies using C1498 AML (a cell line with very rapid leukemia progression) and MLL-AF9 AML (a primary mouse AML). In mice with C1498 AML, MRX2843-treated mice survived longer compared to vehicle-treated mice (28 vs 20 days; p<0.0001) (**Supplemental Figure 1A**). Similarly in mice with MLL-AF9 AML, mice treated with MRX2843 demonstrated prolongation of survival (41 vs 29 days, p<0.0001) (**Figure 1C**).

We hypothesized that the extended survival associated with MRX2843 treatment was specific to MerTK-expressing macrophages *in vivo*. Previous research has demonstrated that MerTK is not expressed in murine (22, 23) or human T cell subsets (24–26), however one study demonstrated that hyper-activated T cells can express MerTK in highly over-activated conditions after prolonged stimulation with anti-CD3/CD28 beads (27). Therefore, we assessed MerTK expression on CD4^+^ and CD8^+^ T cells in leukemic mice treated with vehicle or MRX2843 (and vehicle-treated non-leukemic mice as a control), and did not observe appreciable T cell MerTK expression (**Figure 1D**). Therefore, we determined that any effects of MRX2843 on T cell number or function were likely through alteration of the leukemia microenvironment, rather than attributable to direct cell intrinsic effects on T cells.

To further validate that this survival advantage could be attributable to MerTK inhibition on macrophages in the leukemia microenvironment, we inoculated MLL-ENL or C1498 cells into mice harboring a homozygous *Mertk*-knockout mutation (*Mertk^–/–^*) or their *Mertk*-wildtype littermates (*Mertk^WT^)*, and leukemia-free survival was monitored. Extension of survival was even more striking in these studies. None of the *Mertk^–/–^* mice inoculated with MLL-ENL AML developed leukemia, whereas *Mertk^WT^* littermates had a median survival of 25 days (p<0.001, **Figure 1E**). To ensure that the observed effects were due to blockade of MerTK in macrophages rather than an off target effect of MerTK knockout in non-immune cell subsets, or due to concomitant *Tyro3* deletion which has recently been described in *Mertk^–/–^* mice (28), we performed similar survival studies in conditional knockout model of *Mertk-loxp* ^+/+^ *Cre* ^+^ mice, in which MerTK is knocked-down in only dual MerTK and LysM expressing macrophages. *Mertk-loxp* ^+/+^ *Cre* ^+^ mice inoculated with MLL-ENL AML demonstrated extension of survival of all but one mouse, compared to a median survival of 35 days in *Mertk-loxp* ^-/-^ *Cre* ^-/-^ mice (p<0.001) (**Figure 1F**). In the more rapidly progressive C1498 model, median survival of *Mertk^-/-^* mice was 40 days, versus only 22 days in littermate control *Mertk^WT^* mice (p<0.01, **Supplemental Figure 1B**). Collectively, these results demonstrated that MerTK inhibition of LAMs was sufficient to improve leukemia-free survival *in vivo*.

We next sought to evaluate whether the survival advantage using MRX2843 correlated with altered white blood cell counts (**Figure 1G**). We examined peripheral blood cell populations, including total nucleated cell count by trypan blue exclusion, and peripheral white blood cell populations using flow cytometry. There was no difference in the number of total peripheral blood nucleated cells (TNC), or percentages of granulocytes, monocytes, B cells and NK cells (**Figure 1H**). In MRX2843-treated mice, the percentage of peripheral blood CD3^+^ T cells (13%) was higher than vehicle-treated mice (6%, p<0.05) (**Figures 1I, J**). Prior studies using MRX2843 in preclinical models demonstrate no alterations in other blood cell indices (white blood cell count, red blood cell count, or platelet count) (21).

We also evaluated the effect of MRX2843 on myeloid populations in the spleen and BM of mice inoculated with MLL-ENL AML and treated with MRX2843 or vehicle (**Figure 1G**). Vehicle-treated non-leukemic mice were used for comparison. The total number of nucleated cells in the spleen and BM were similar between the three groups (**Figure 1J**). Within the myeloid cell lineage, MerTK is commonly expressed on mature macrophages, including leukemia-associated macrophages (LAMs; CD11b^+^CD11c^+^Gr-1^-^), but not on monocytes (CD11b^+^CD11c^-^Gr-1^-^SideScatter^lo^), granulocytes (CD11b^+^CD11c^-^Gr-1^+^SideScatter^hi^), or myeloid-derived suppressor cells/myeloid progenitors (MDSCs; CD11b^+^CD11c^-^Gr-1^intermediate^SideScatter^hi^) (19). Gr-1^+^ monocytes (CD11b^+^CD11c^-^Gr-1^+^SideScatter^lo^) have variable MerTK expression. These myeloid populations (**Supplemental Figure 1B**) were analyzed for alterations with MRX2843 treatment. LAMs were the only population that were significantly decreased with MRX2843 treatment within both the spleen (p<0.05) and marrow (p<0.01) (**Figure 1K**; **Table 1**). There was no significant difference in the number of Gr-1^-^ monocytes, granulocytes, or MDSCs/myeloid progenitors between treatment groups (**Table 1**; **Supplemental Figures 1C, E, F**). Within the spleen, MRX2843 treatment yielded a significant decrease of Gr-1^+^ monocytes, though there was no difference within the marrow (**Table 1**; **Supplemental Figure 1D**).

**Table 1:** Cell counts with MerTK inhibition.

				Spleen			Marrow		
				No leukemia	AML + vehicle	AML + MRX2843	No leukemia	AML + vehicle	AML + MRX2843
Total nucleated cells (x10^6^/dL)				12.2 ± 16.3	18.1 ± 13.7	10.0 ± 8.5	8.6 ± 6.0	10.6 ± 7.9	4.4 ± 3.0
Myeloid (x10^5^ cells/dL)	LAMs			1.4 ± 0.5	1.9 ± 0.8	0.9 ± 0.6	0.7 ± 0.4	1.3 ± 0.7	0.4 ± 0.1
	MDSCs/Myeloid Precursors			0.3 ± 0.1	0.3 ± 0.5	0.1 ± 0.1	1.4 ± 0.9	0.9 ± 0.6	0.8 ± 0.7
	Gr-1^-^ Monocytes			14.3 ± 0.5	1.6 ± 1.4	1.0 ± 0.6	0.9 ± 0.4	0.7 ± 0.4	0.6 ± 0.4
	Granulocytes			1.2 ± 0.5	1.7 ± 1.3	0.7 ± 0.3	29.8 ± 14.3	12.9 ± 10.1	21.6 ± 15.7
	Gr-1^+^ Monocytes			1.1 ± 0.5	2.0 ± 1.5	0.7 ± 0.4	9.9 ± 3.0	6.9 ± 3.7	8.9 ± 6.4
LAMs (x10^4^ cells/dL)	PD-L1^+^			6.9 ± 2.6	11.9 ± 4.6	4.9 ± 3.1	4.0 ± 2.8	9.8 ± 4.7	2.2 ± 1.3
	PD-L2^+^			0.7 ± 0.4	3.6 ± 1.8	0.6 ± 0.4	0.5 ± 0.3	3.1 ± 2.0	0.2 ± 0.1
T cells (x10^4^ cells/dL)	CD4^+^			9.3 ± 2.3	8.6 ± 3.9	8.2 ± 3.3	4.4 ± 2.9	4.8 ± 2.7	3.8 ± 2.2
	CD8^+^			9.7 ± 4.0	10.4 ± 2.0	9.3 ± 4.4	10.1 ± 7.0	7.5 ± 5.0	7.5 ± 6.3
	CD4^+^ Tim-3^+^ PD-1^+^			0.2 ± 0.2	2.7 ± 1.6	0.2 ± 1.8	0.7 ± 0.6	3.9 ± 2.8	0.7 ± 0.3
	CD8^+^ Tim-3^+^ PD-1^+^			0.3 ± 0.2	1.2 ± 0.8	0.2 ± 1.4	0.6 ± 0.4	6.0 ± 6.8	0.4 ± 0.6

Total viable cell numbers within the spleen and marrow of mice from each treatment groups. Numbers represent means ± standard deviations. LAMs, Leukemia associated macrophages; MDSCs, myeloid derived suppressor cells.

### MerTK inhibition skews LAM polarization

**Figure 2:**
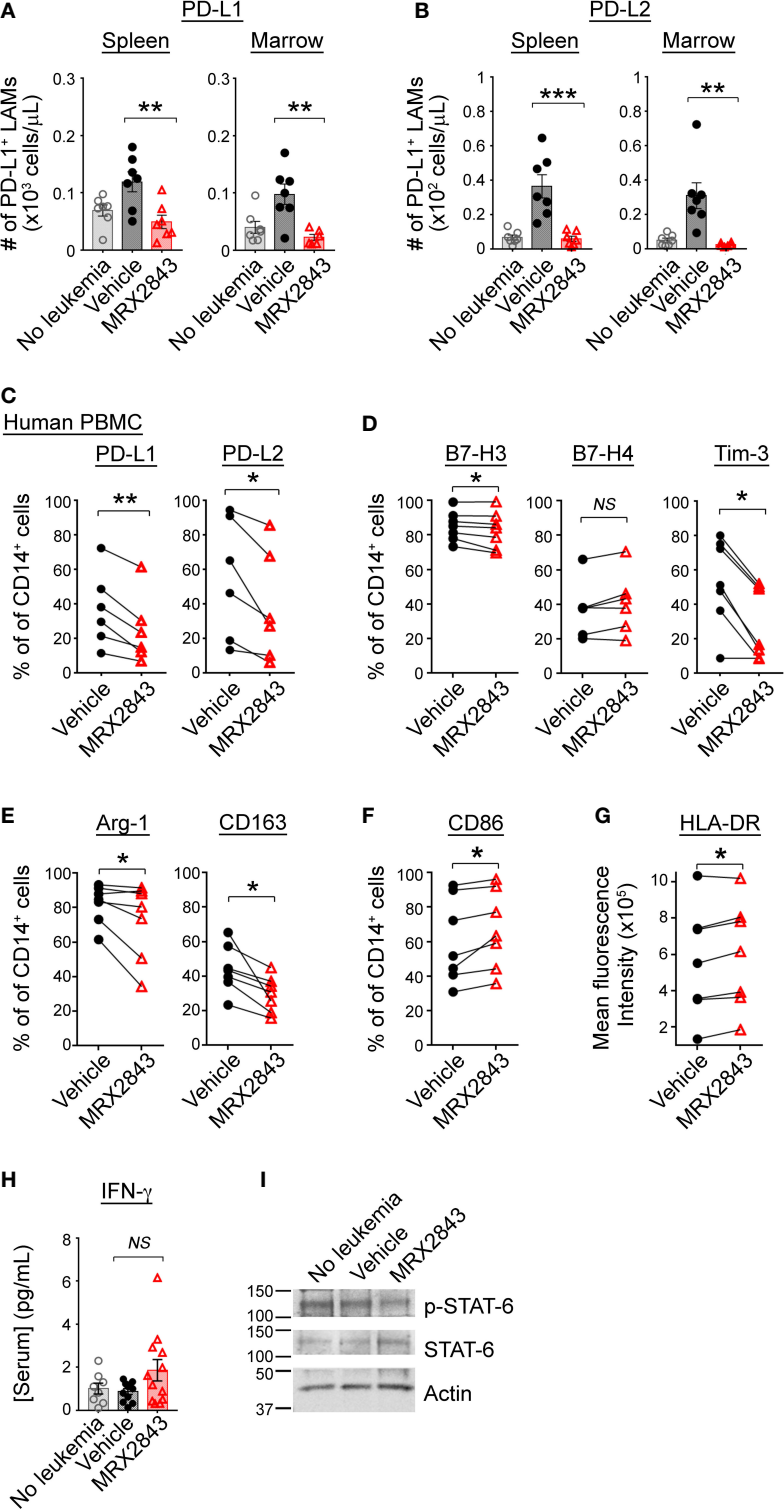
MerTK inhibition skews macrophage polarization in the leukemia microenvironment. **(A, B)** As described in **Figure 1G**, C57BL/6 mice were inoculated with syngeneic MLL-ENL AML and treated daily with MerTK inhibitor MRX2843 (red triangle) or vehicle (saline; black circle); mice not inoculated with leukemia are shown as controls (gray open circle). Three weeks after inoculation, spleens and marrow were harvested and assessed by flow cytometric analysis. **(A)** Quantification of PD-L1 expressing LAMs in the spleen and marrow. **(B)** Quantification of PD-L2 expressing LAMs in the spleen and marrow. **(C–G)** Macrophages derived from human peripheral blood mononuclear cells (PBMC) co-cultured with Kasumi-1 AML cells and MRX2843 or vehicle were analyzed by flow cytometry. Percentage of cultured macrophages expressing **(C)** PD-L1 and PD-L2, and **(D)** B7-H3, B7-H4 and Tim-3, **(E)** Arginase-1, CD163, and **(F)** CD86. **(G)** Flow cytometric quantification of HLA-DR mean fluorescence intensity of co-cultured macrophages. **(H)** Serum from mice described in **Figure 1G** was subjected to Luminex for interferon-gamma (IFN-γ). **(I)** Murine bone marrow derived macrophages (BMDMs) were co-cultured with MLL-ENL AML cells and macrophage whole cell lysates were analyzed by immunoblot. Representative immunoblot showing phosphorylated STAT6 (p-STAT6) and total STAT6. Actin was used as a loading control. **(A, B, H)** were analyzed using 1-way ANOVA. **(B–G)** were analyzed using student’s t-test (*p<0.05, **p<0.01, ***p<0.001, NS, not significant).

We previously reported an effect of MerTK inhibition on PD-L1 and PD-L2 expression in acute lymphoblastic leukemia (ALL) (21), however, given the vast differences between these diseases and the microenvironments they create within the spleen and BM, we evaluated LAM PD-L1 and PD-L2 expression in mice inoculated with MLL-ENL AML treated with MRX2843 (or vehicle), and non-leukemic mice (control). The leukemia microenvironment contained significantly more PD-L1 (spleen: p<0.01; BM: p<0.01) and PD-L2 (spleen: p<0.001; BM: p<0.01) expressing LAMs in vehicle-treated mice compared to MRX2843-treated mice (**Figures 2A, B**; **Table 1**; **Supplemental Figure 2A**). When evaluating the median fluorescence intensity of PD-L1 and PD-L2 expression on LAMs, we observed an up-field shift in the PD-L1 curve indicating globally increased expression on all LAMs (**Supplemental Figure 2B**), whereas PD-L2 upregulation occurred in a discrete subpopulation of LAMs (**Supplemental Figure 2C**). Similar PD-L1 and PD-L2 changes were observed in mice with C1498 AML treated with MRX2843 or vehicle (**Supplemental Figures 2D, E**).

To evaluate whether human macrophages are affected similarly in response to MRX2843, donor PBMC-derived macrophages were co-cultured with Kasumi-1 human AML cells for 48 hours. Prior to co-culture, donor macrophages expressed abundant MerTK (**Supplemental Figure 2F**). Of note, UV-exposed AML cells were positive for PtdSer expression (Annexin-V staining, **Supplemental Figure 2G**), but demonstrated very little irreversible apoptosis (i.e., propidium iodide uptake). After 48 hours of co-culture, there was broad variation of PD-L1 and PD-L2 expression on vehicle-treated donor macrophages, however MRX2843-treatment significantly decreased expression of PD-L1 by 23% (p<0.01) and PD-L2 by 31% (p<0.05) (**Figure 2C**).

Additional co-inhibitory receptors that portend a M2-like phenotype were analyzed on human macrophages co-cultured with AML cells. Macrophage expression of B7-H3 was minorly decreased (p<0.05) and B7-H4 was unchanged when MerTK was inhibited (**Figure 2D**). However, there was a striking 47% decrease in Tim-3 expression on macrophages co-cultured with AML cells and treated with MRX2843, compared to vehicle treatment (p<0.01) (**Figure 2D**). Similarly, M2-like markers CD163 (decreased 37%, p<0.05) and Arginase-1 (decreased 27%, p<0.05) were significantly downregulated in MRX2843-treated macrophages compared to vehicle treatment (**Figure 2E**). Furthermore, M1 macrophage activation was observed *via* a 111% increased cell surface expression of CD86 (p<0.05) (**Figure 2F**) and increased mean fluorescence intensity of HLA-DR (p<0.05) (**Figure 2G**).

Upregulation of cell surface PD-L1 and PD-L2 has been associated with exposure to IFN-γ (29–32), therefore we assessed serum IFN-γ concentrations in MRX2843 or vehicle-treated mice by Luminex. Conversely, we observed a trend of slightly higher serum IFN-γ concentrations from the MRX2843-treated mice compared to vehicle-treated mice (vehicle: 0.9pg/ml; MRX2843: 1.9pg/ml, p=0.1) though overall serum IFN-γ was very low for all groups (**Figure 2H**).

Given the previous association between IL-4/STAT6 signaling, M2-like polarization and upregulation of PD-L1, PD-L2 and Tim-3 (29, 33–35), we assessed the murine serum samples for IL-4. Serum IL-4 concentrations were evaluated, but demonstrated very low mean concentrations (<1pg/ml) in all treatment groups (not shown). Based on our previous leukemia cell line data demonstrating a correlation between STAT6 and MerTK phosphorylation (17), we assessed protein lysates from bone marrow derived macrophages (BMDMs) cultured with (or without) AML cells by immunoblot. MRX2843-treated BMDM demonstrated consistently diminished STAT6 phosphorylation compared to vehicle (**Figure 2I**), providing a possible mechanism for the observed alterations in polarization. SOCS1 and SOC3 have previously been associated with MerTK immunomodulation (36) through inhibition of JAK/STAT, however we did not detect a change in SOCS mRNA levels between treatment groups (**Supplemental Figure 2H**).

### MerTK inhibition leads to production of cytokines known to activate T cells

**Figure 3:**
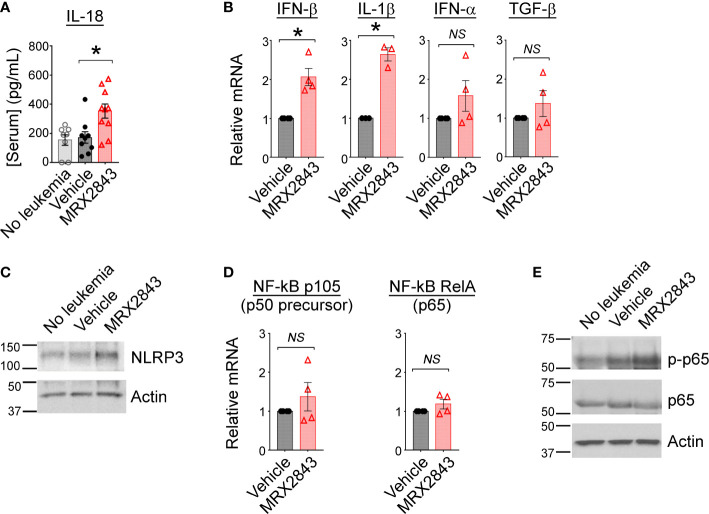
Inhibition of MerTK results in M1 cytokine production through activation of NF-κB. **(A)** Serum from mice described in **Figure 1G** was subjected to Luminex for interleukin-18 (IL-18); analyzed using 1-way ANOVA. **(B)** BMDM were co-cultured with MLL-ENL cells for 12 hours in the presence of MRX2843 or vehicle, then mRNA was harvested from adherent macrophages. Quantitative real time-PCR was performed, normalized to GAPDH expression, and then gene expression fold-change relative to vehicle-treated samples was calculated. Analyzed using Student’s t-test. **(C)** As described in **Figure 2I**, whole cell lysates from BMDM were analyzed by immunoblot. Representative immunoblot demonstrating NLRP3, a component of the inflammasome, is shown; Actin is used as a loading control. **(D)** RT-PCR was performed as described in **(B)** Gene expression fold-change of the p105 (precursor to p50) and RelA (p65) components of NF-κB relative to vehicle-treated samples was calculated. Analyzed using Student’s t-test. **(E)** Representative immunoblot demonstrating phosphorylated p65/RelA and total p65/RelA; Actin was used as a loading control. (*p<0.05, NS, not significant).

To further assess the immunological implications of efferocytosis/MerTK inhibition with MRX2943, we evaluated the impact of LAM MerTK signaling using multiplex cytokine Luminex panel. IL-18, indicative of M1 macrophage activation, was significantly increased in MerTKI-treated mice (352.7pg/ml) compared to vehicle-treated controls (171.6pg/ml, p<0.05) (**Figure 3A**). We also observed a trend toward higher serum concentrations of IL-6, a cancer-promoting cytokine, in some vehicle-treated mice (379.1pg/ml) compared to MRX2843-treated mice (45.4pg/ml, p=0.3) (**Supplemental Figure 3A**). The following cytokines/chemokines demonstrated no significant difference between MRX2843-treated mice and controls: IL-10, IL-12p70, IL-27, MCP-3 (**Supplemental Figure 3B**). Additionally, serum levels of IL-1β and IL-2 were evaluated, however, the mean concentration of these cytokines in the serum were <1pg/ml in all treatment groups (not shown).

To evaluate cytokine production that may be produced locally in the leukemia microenvironment, but not be detected peripherally in the serum by Luminex, cultured BMDMs were co-cultured with AML cells and treated with MRX2843 or vehicle. After 12 hours, harvested BMDMs were evaluated *via* RT-qPCR. Compared to vehicle-treated controls, IFN-β transcripts were increased 2.1-fold in MRX2843-treated BMDMs (p<0.05), and IL-1β mRNA was increased 2.6-fold in MRX2843-treated samples (p<0.05) (**Figure 3B**). There was no significant difference between vehicle and MRX2843-treated groups for INF-α or TGF-β. IL-10 and IL-12 mRNA levels were not consistently detectable in these samples (not shown) despite testing of two distinct commercially available validated primer pairs each. Additionally, co-cultured macrophages did not demonstrate detectable amounts of IFN-γ mRNA (not shown), suggesting that the trend seen *in vivo* in murine serum samples were not directly derived from LAMs but from other downstream effector cells in the leukemic microenvironment as consequence of MerTK inhibition in LAMs.

### MerTK inhibition alters NF-κB activation

Given the increased levels of IL-18 and IL-1β with MRX2843-treatment, we evaluated for components of the inflammasome in BMDMs co-cultured with AML cells. Increased NLRP3 was observed by immunoblot analysis when BMDMs were co-cultured with AML cells and MRX2843, compared to vehicle-treatment (**Figure 3C**). Previously, inhibition of MerTK signaling has been associated with increased NF-κB signaling (36), possibly through differential expression of the p50 subunit (37). We assessed mRNA levels of p105 (p50 precursor) and RelA (p65) in BMDM co-cultured with AML cells and MRX2843 or vehicle, however there was no difference in transcripts (**Figure 3D**). However, by immunoblot analysis of BMDMs, p65 phosphorylation was increased after MRX2843 treatment compared to vehicle (**Figure 3E**). Therefore, in this model of AML, MRX2843-mediated immune activation was associated with NF-κB phosphorylation, rather than altered ratios of p50:p65 transcription.

### Inhibition of MerTK on LAMs activates T cells

**Figure 4:**
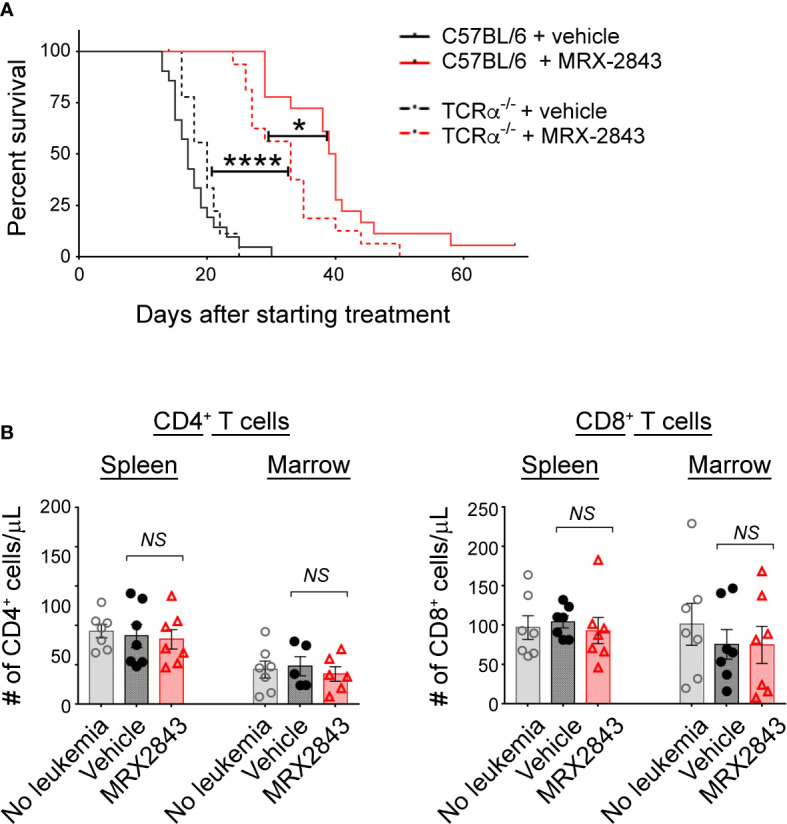
MerTK Inhibition on LAMs effects T cells in the leukemia microenvironment. As described in **Figure 1G**, mice were inoculated with AML and treated with MRX2843 or vehicle. **(A)** Cohorts of mice lacking the α chain of the T cell receptor (TCRα^-/-^) were inoculated with AML and treated equivalently to those described in **Figure 1B**. Symptom-free survival was recorded; Kaplan-Meier curve of TCRα^-/-^ mice treated with MRX2843 (dashed red line; n = 17) or vehicle (dashed black line, n = 9) with the superimposed wild-type C57BL/6 mice described in **Figure 1B** treated with MRX2843 (solid red line) and vehicle (solid black line). **(B)** Three weeks after inoculation, harvested spleen and marrow from MRX2843 or vehicle-treated mice were analyzed by flow cytometry. Quantification of CD4+ and CD8+ T cells was analyzed using 1-way ANOVA. (*p<0.05, ****p<0.0001, NS, not significant).

To assess whether the prolonged survival observed with MerTK inhibition in the leukemia microenvironment is dependent on T cell function, we performed survival studies in leukemic mice that lack the T cell receptor alpha chain (TCRα^-/-^) rendering them deficient in α/β T cells. Vehicle-treated leukemic C57BL/6 mice with a wild-type TCR alpha chain (17 days) and TCRα^-/-^ mice (20 days) had equivalent median survival (**Figure 4A**). As show in **Figure 1B**, leukemic C57BL/6 mice with a wild-type TCR alpha chain treated with MRX2843 demonstrated a statistically prolonged survival compared to vehicle treatment. MRX2843 treatment extended survival in TCRα^-/-^ leukemic mice (33 days; p<0.0001) compared to vehicle treatment; however, survival was significantly diminished compared to C57BL/6 MRX2843-treated mice (p<0.05), demonstrating that immune-mediated clearance of AML with MRX2843 treatment is optimal when α/β T cells are present.

### MerTKI indirectly activates T cells in the leukemia microenvironment

To further explore this observation, we evaluated for alteration of CD4^+^ and CD8^+^ T cell infiltration into the leukemia microenvironment. However, the total number of CD4^+^ and CD8^+^ T cells in the spleen and BM were not different between treatment groups (**Table 1**; **Figure 4B**).

**Figure 5:**
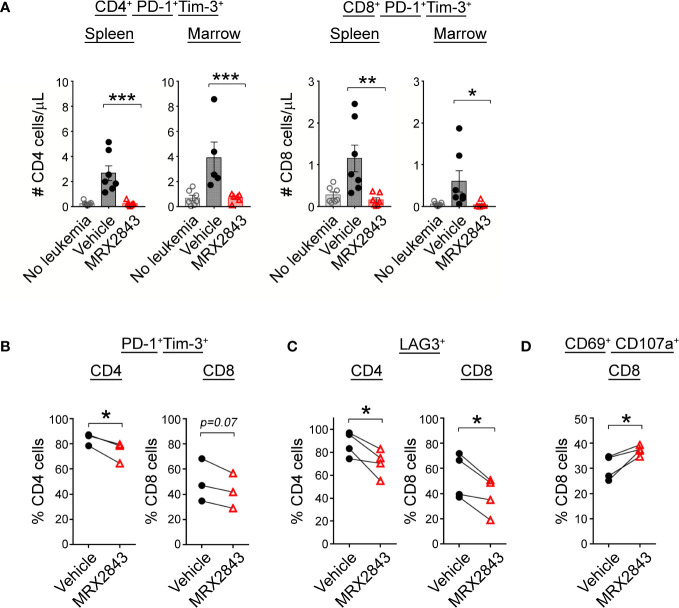
MerTK inhibition boosts T cells activation. **(A)** As described in **Figure 1G**, harvested spleen and marrow were analyzed by flow cytometry. Quantified CD4^+^ and CD8^+^ T cells co-expressing PD-1 and Tim-3 were analyzed using 1-way ANOVA. **(B)** Mixed human leukocyte co-cultures containing macrophages treated with MRX2843 or vehicle, AML cells and T cells were analyzed by flow cytometry. Percentage of cultured CD4^+^ and CD8^+^ T cells expressing **(B)** PD-1 and Tim-3, **(C)** LAG3, and **(D)** CD69 and CD107a. Analyzed using Student’s t-test. (*p<0.05, **p<0.01, ***p<0.001).

To assess the downstream effects that LAM MerTK inhibition had on T cell function in the leukemia microenvironment, we assessed checkpoint receptor expression predictive of T cell hypofunction and/or exhaustion. There were significantly less PD-1^+^Tim-3^+^ expressing CD4^+^ cells in the spleen (p<0.001) and BM (p<0.01) of MRX2843-treated mice compared to vehicle-treated mice (**Figure 5A**; **Table 1**). The same pattern of fewer PD-1^+^Tim-3^+^ expressing CD8^+^ cells in the spleen (p<0.01) and BM (p<0.05) was observed in MRX2843-treated mice compared to vehicle-treated mice (**Figure 5A**; **Table 1**).

To validate these findings in human T cells, we assessed CD4^+^ and CD8^+^ T cells co-cultured with AML-exposed macrophages in the presence of MRX2843 or vehicle. In MRX2843-treated samples, we observed a 12% mean reduction of PD-1^+^Tim-3^+^ expressing CD4^+^ T cells (p<0.05), and a 14% reduction in CD8^+^ PD-1^+^Tim-3^+^ cells compared to vehicle-treatment (p=0.07) (**Figure 5B**). In these co-cultures, we also assessed expression of checkpoint receptor LAG3 (**Figure 5C**), which was reduced by 29% on CD4^+^ T cells (p<0.05) and by 19% in CD8+ T cells in MRX2843-treated co-cultures compared to vehicle (p<0.05).

To assess the impact of altered macrophage polarization on CD8^+^ T cell activation, co-expression of activation marker CD69 and degranulation marker CD107a was assessed (**Figure 5D**). In MRX2843-treated mixed lymphocyte co-cultures, there was a 23% increase in CD69^+^CD107a^+^ co-expression compared to vehicle-treated co-cultures (p<0.05).

## Discussion

**Figure 6:**
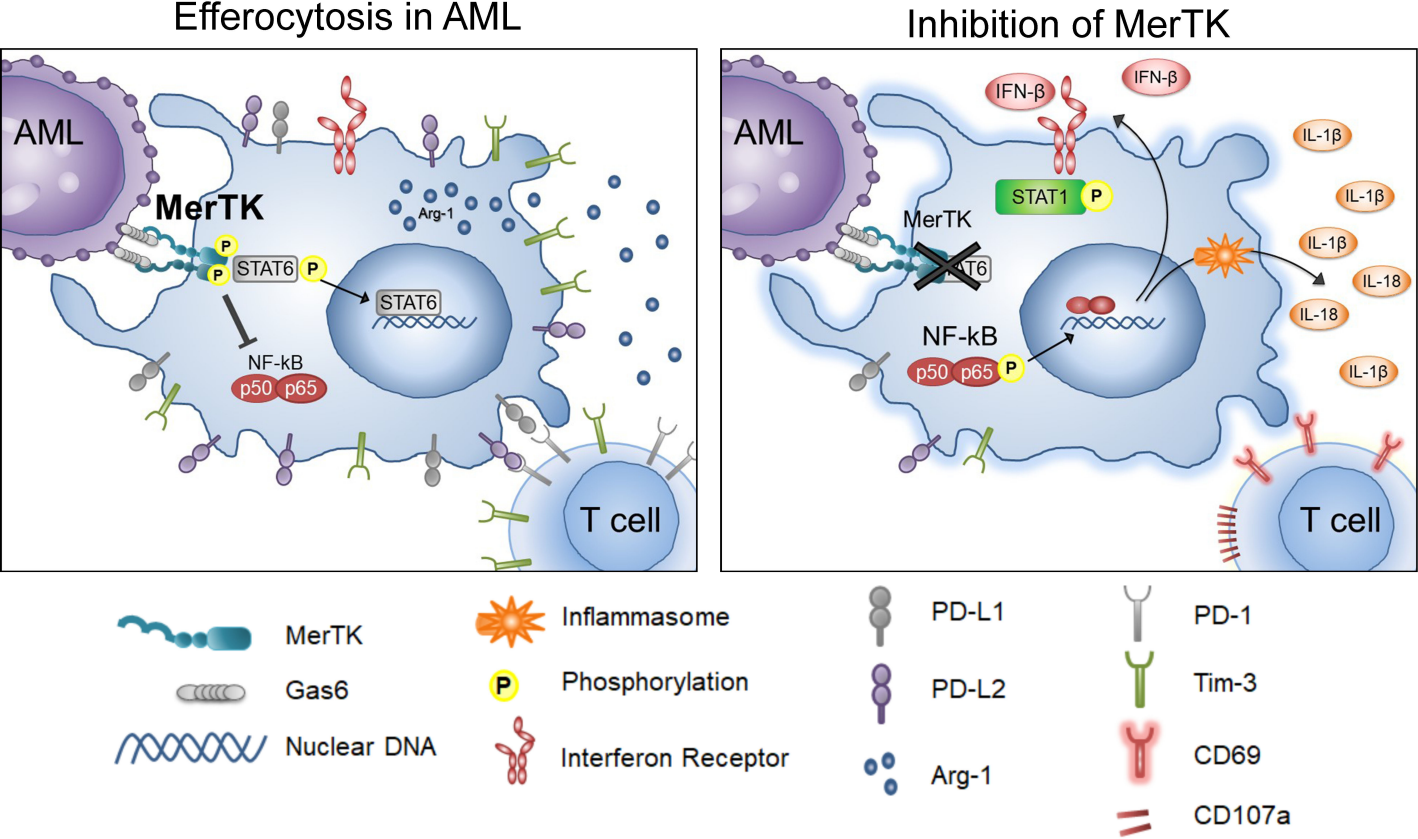
Inhibition of Efferocytosis through MerTK in AML. Graphical depiction of the findings in these studies, including the activities of leukemia-associated macrophages when MerTK engages in efferocytosis in AML (left), and when efferocytosis through MerTK it is inhibited in AML (right).

MerTK inhibition has been evaluated in various tumor models for its cancer cell intrinsic properties (16–19, 38, 39), directing the development and testing of MerTK-targeted small molecule inhibitors. Here, we mechanistically explored the action of MRX2843, a clinically available MerTKI, on macrophage polarization in the leukemia microenvironment (**Figure 6**). Recent research highlights the profound immunosuppressive effects of intratumoral myeloid cells such as TAMs/LAMs, including the ability to render chimeric antigen receptor T cells and other immunotherapy ineffective (12, 40–42). Given that PtdSer, the ligand for efferocytosis, is overexpressed on cancer cells due to high cell turnover and poor cell membrane maintenance, we posited that efferocytosis is one of the primary causes of macrophage-mediated immunosuppression within the leukemia microenvironment. Therefore, targeting efferocytosis through MerTK may help reverse immunosuppression in the leukemia microenvironment. Here we tested the activity of MRX2843, an orally-active small molecule as immunotherapy with rapid on/off activity of competitive inhibitor, in the AML tumor microenvironment.

Targeting efferocytic macrophages within the spleen and marrow leukemia microenvironments, we demonstrated that MRX2843 decreased macrophage immunosuppressive features including a reduction in M2-like markers PD-L1, PD-L2, Tim-3, CD163, and Arginase-1. Additionally, we observed that MRX2843 treatment increased expression of M1 markers CD86 and HLA-DR, and the production of IFN-β, IL-1β and IL-18, cytokines known to stimulate activation of T cells. Collectively, these observations demonstrate macrophage repolarization from M2-like tumor-permissive phenotype toward a M1 anti-tumor activation, and prolongation of leukemia-free survival.

The mechanism of this repolarization is likely associated with the inhibition of MerTK downstream signaling. STAT6 is known to augment coinhibitory receptor ligand expression and M2 polarization (29, 43, 44). We previously reported that in leukemia cells, MerTK inhibition using short-hairpin RNA and MerTKIs decreased STAT6 phosphorylation (17, 18). Here, we found that MRX2843 treatment similarly decreased STAT6 phosphorylation in leukemia-exposed macrophages, providing a plausible mechanism by which MerTK inhibition leads to macrophage repolarization from cancer-promoting toward a classically activated phenotype. Additionally, production of T cell and NK cell activating cytokines (45) likely occurred due to inflammasome assembly as a result of NF-κB activation.

In this study, we demonstrated that LAM repolarization associated with MRX2843 treatment had additional influence on the leukemia microenvironment. MRX2843 altered T cell checkpoint receptor expression and activation markers. However, we conclude that this effect was likely indirect given that that vast majority of T cells did not express MerTK. Though MRX2843 treatment of LAMs alone (in the absence of α/β T cells) was sufficient to prolong survival in leukemic mice, it is notable that when host T cells were present, MRX2843 had a more potent effect on survival prolongation. These data suggest that this therapeutic approach might be beneficial to boost T cell-directed immunotherapy in immunologically cold (or macrophage infiltrated) leukemias or tumors. Although our work focused on how LAM efferocytosis effected T cells, future work may evaluate how MRX2843 (and similar MerTKIs) effect NK cell infiltration and cytolysis in the leukemia/tumor immune microenvironment.

Taken together, these studies demonstrate how inhibition of efferocytosis could be harnessed to alter the microenvironment from tumor-permissive to immune responsive in leukemia. Furthermore, given that a wide variety of cancers are known to express MerTK, including nearly 80-90% of AML patient samples (17), MerTK inhibitors could potentially have a dual cell intrinsic effect on AML cells and antitumor immune response through LAMs. Future work will focus on how effectively MerTK inhibitors can serve these dual purposes.

## Data availability statement

The raw data supporting the conclusions of this article will be made available by the authors, without undue reservation.

## Ethics statement

The animal study was reviewed and approved by Institutional Animal Care and Use Committee (IACUC), CU-AMC.

## Author contributions

JC, KA, LP, AJ, MV, and AL-S designed and performed experiments and analyzed data. HE, SF, XW, and DG analyzed data. JC, KA, LP, AJ, HE, SF, XW, MV, and AL-S wrote the manuscript. All authors contributed to the article and approved the submitted version.
