# Supplementary material for: Inhibiting efferocytosis reverses macrophage-mediated immunosuppression in the leukemia microenvironment
Source: Front Immunol. 2023 Mar 7;14:1146721. doi: 10.3389/fimmu.2023.1146721 (PMC10027704; doi:10.3389/fimmu.2023.1146721)
Supplement: Supplementary file 1 [file DataSheet_1.pdf]

Supplemental Methods Table I.

IMMUNOBLOT ANTIBODIES

Antigen	Manufacturer	Catalog Number
Actin	Invitrogen	#MA515739HRP
MerTK	R&D Systems	AF591
Phospho-STAT6	Cell Signaling	565546
STAT6	Cell Signaling	9362S
NLRP3	Cell Signaling	15101S
Phospho-P65	Cell Signaling	3033S
P65	Cell Signaling	8242S
goat anti-rabbit IgG-HRP	Biorad	1706515

FLOW CYTOMETRY ANTIBODIES

Mouse:

Antigen	Clone	Manufacturer	Catalog Number
CD45	30-F11	Biolegend	103138
Ly-6G/C (Gr-1)	RB6-8C5	Biolegend	108412
CD11b	M1/70	BD Biosciences	557657
CD11c	N418	Biolegend	117318
PD-L1	10F.9G2	Biolegend	124315
CD273 (PDL2)	TY25	Biolegend	107205
CD3	17A2	Biolegend	100216
CD4	RM4-5	eBioscience	25-0042-82
CD8	53-6.7	eBioscience	47-0081-81
B220	RA3-6B2	eBioscience	61-0452-82
CD161 (NK1.1)	PK136	Biolegend	108735
PD-1	RMP1-30	Biolegend	109112
TIM3	RMT3-23	eBioscience	12-5870-82

Human:

Antigen	Clone	Manufacturer	Catalog Number
CD14	MΦP-9	BD Biosciences	557832
CD11c	3.9	Biolegend	301607
CD16	ebio16(CB16)	Invitrogen	56016842
PDL1	29E.2A3	Biolegend	329713
CD273 (PDL2)	24f.10c12	Biolegend	329605
MerTK	125518	R&D	FAB8912A
B7-H3 (CD276)	7-517	Invitrogen	25276941
B7-H4	MI-H43	BD Optibuild	742960

TIM3	F38-2E2	Biolegend	345052
CD163	RM3/1	Biolegend	333615
Arginase-1	A1exF5	Invitrogen	12369782
CD86	IT2.2	Biolegend	305408
CD3	UCHT1	Biolegend	58003842
CD4	SK3	BD Biosciences	563550
CD8a	OKT8	Thermo-Fisher	56008642
PD-1	EH12.2H7	Biolegend	329924
Tim3	F38-2E2	Biolegend	345052
Lag3	11C3C65	Biolegend	369314
CD107a	H4A3	Biolegend	328610
CD69	FN50	Biolegend	310932

TAQMAN PRIMERS (Thermo Scientific)

Target	Catalog Number
GAPDH	Mm99999915
IFNβ	Mm00439552
IL-1b	Mm00434228
IFNα	Mm03030145
TGFb	Mm01178820
SOCS1	Mm00782550
SOCS3	Mm00545913
RelA (p65)	Mm00501346
p105 (NFkB)	Mm00476361
IL-10	Mm01288386
IL-10	Mm00439614
IL-18	Mm00434225
IL-18	Mm00434226
IL-12b	Mm01288989
IL-12b	Mm00434174
