# Supplementary material for: Inhibiting efferocytosis reverses macrophage-mediated immunosuppression in the leukemia microenvironment
Source: Front Immunol. 2023 Mar 7;14:1146721. doi: 10.3389/fimmu.2023.1146721 (PMC10027704; doi:10.3389/fimmu.2023.1146721)
Supplement: Supplementary file 2 [file DataSheet_2.pdf]

Supplemental Figure 1

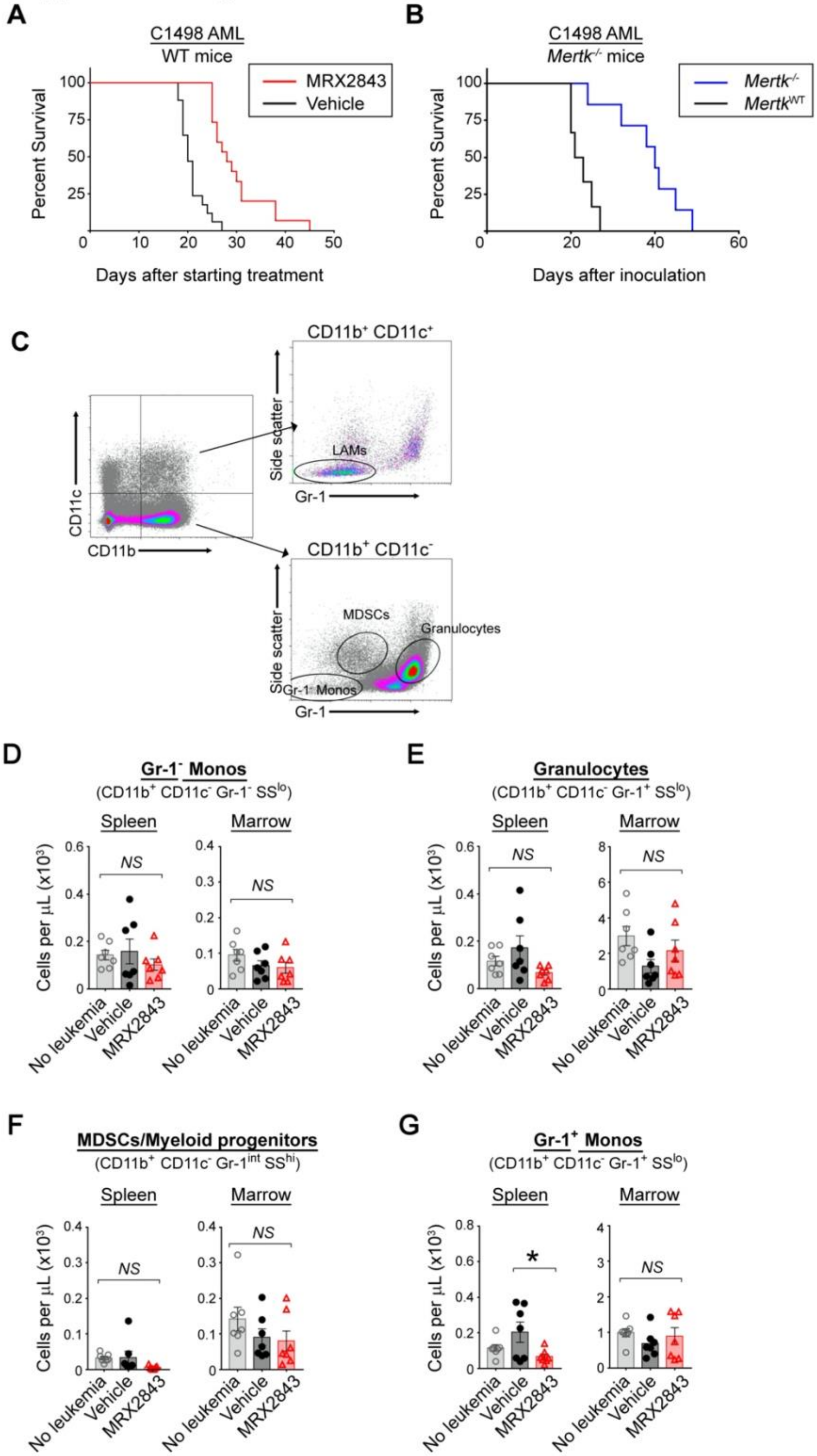

Supplemental Figure 1: MerTKI inhibition specifically affects leukemia associated macrophages. (A-B) Mice inoculated with $5x10^3$ C1498 AML cells, and weight loss and health score were monitored as surrogates for disease burden; Kaplan-Meier survival analysis measured leukemia-free survival. (A) Starting three days after C1498 inoculation, C57Bl/6 mice began daily treatment with MerTK inhibitor MRX2843 (60mg/kg; red line, n =18) or an equivalent volume of vehicle (PBS; black line, n =21) in three independent replicates. (B) C1498 cells were inoculated into *Mertk*$^{-/-}$ mice (blue line, n =6) or littermate wild-type controls (*MerTK*^{WT}; n =7, black line) by tail vein injection in two independent replicates. Survival was analyzed using log-rank test, comparing MerTK inhibited (MRX2843 or *Mertk*$^{-/-}$) to controls (vehicle or *MerTK*^{WT}). (C-G) C57BL/6 mice were inoculated with syngeneic MLL-ENL AML and treated daily with MerTK inhibitor MRX2843 as described in Figure 1G. (C) Flow cytometric dot plots demonstrate gating strategy of each myeloid cell type. (D-G) Three weeks after inoculation, spleens and marrow were harvested and Gr-1$^-$ monocytes, granulocytes, MDSCs/myeloid progenitors, and Gr-1$^+$ monocytes were quantified by flow cytometry and analyzed using 1-way ANOVA. (*$p<0.05$, NS = not significant)

Supplemental Figure 2

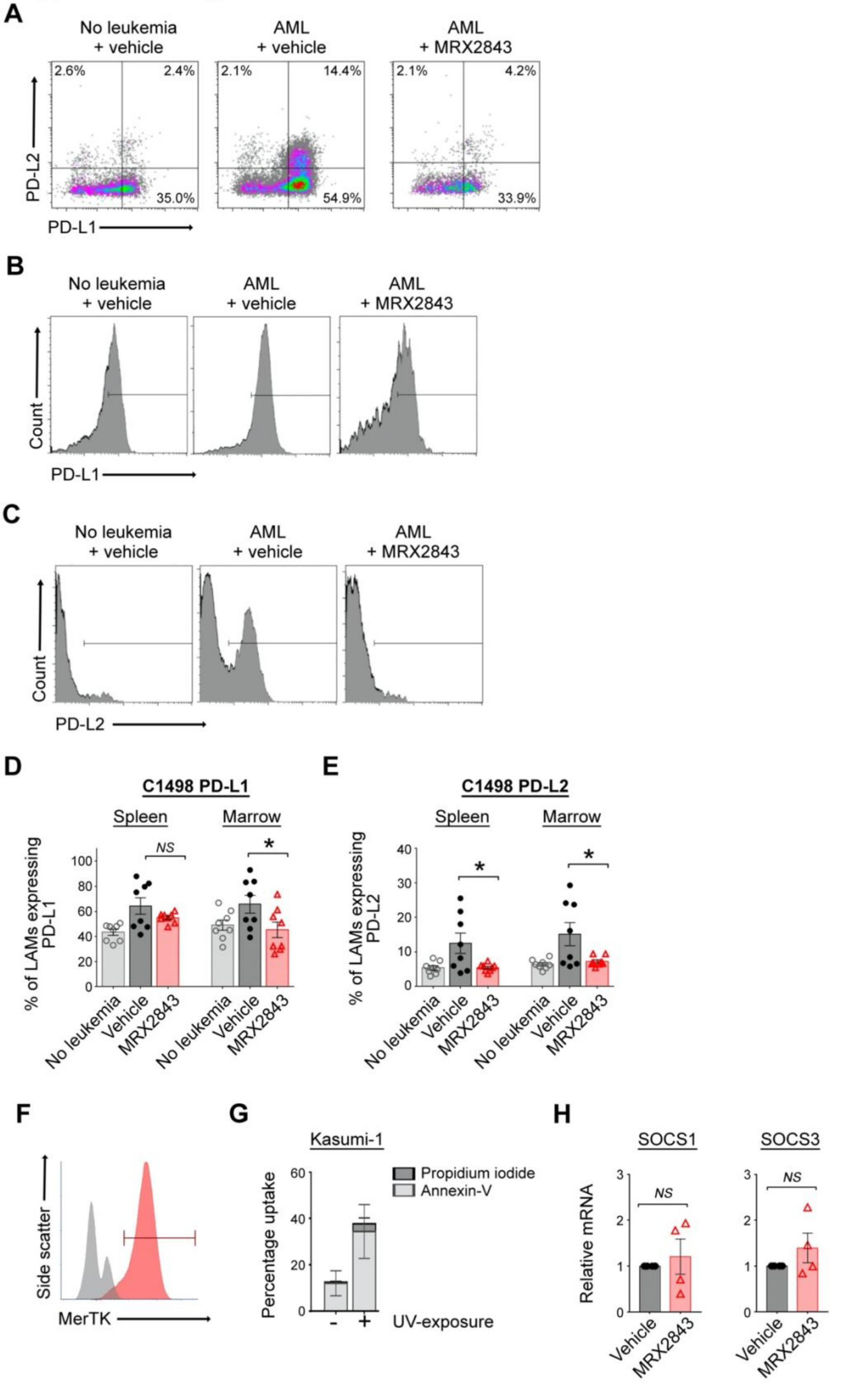

Supplemental Figure 2: Inhibition of MerTK in AML decreases LAM PD-L1 and PD-L2 expression in vivo. (A-C) Syngeneic C57BL/6 mice inoculated with MLL-ENL AML were treated with 60mg/kg of MerTK inhibitor MRX2843 once daily or an equivalent volume of vehicle (saline) for three weeks. Harvested spleens and bone marrow were analyzed by flow cytometry for expression of PD-L1 and PD-L2 on LAMs. (A) Representative dot plots of PD-L1 and PD-L2 expression in LAMs. (A-B) Representative histograms of (B) PD-L1 expression and (C) PD-L2 expression in each treatment group. (D-E) Starting three days after inoculation with C1498 AML, C57BL/6 mice were treated with 60mg/kg of MerTK inhibitor MRX2843 once daily or an equivalent volume of vehicle (saline) for three weeks. Harvested spleen and bone marrow were analyzed by flow cytometry for expression of (D) PD-L1, and (E) PD-L2 on LAMs in the spleen and marrow. Analyzed by 1-way ANOVA (*$p<0.05$, NS = not significant) (F) Representative histograms of MerTK expression on human cultured macrophages (red) compared to isotype control (gray). (G) Kasumi-1 AML cells with and without exposure to UV were stained for Annexin-V and Propidium Iodide, and analyzed by flow cytometry. (H) Murine bone marrow derived macrophages were co-cultured with apoptotic MLL-ENL cells for 48 hours and quantitative real time-PCR was performed. Gene expression fold-change of SOCS1 and SOCS3 relative to vehicle-treated samples was calculated, and analyzed using student’s t-test.

Supplemental Figure 3

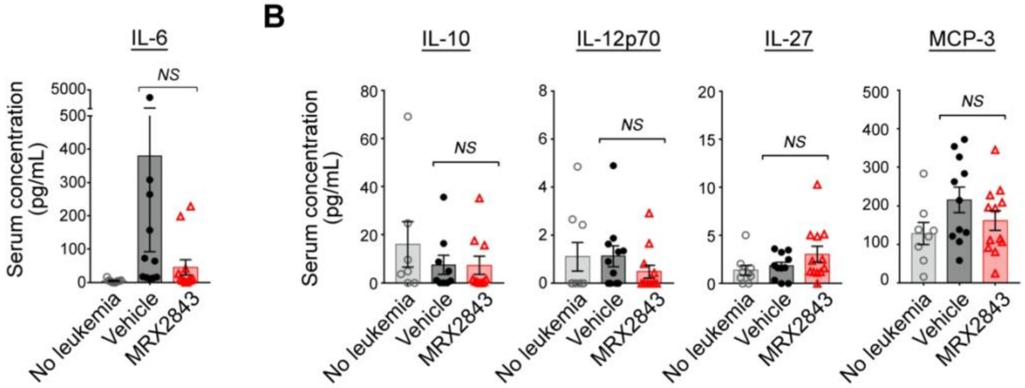

Supplemental Figure 3: Cytokine assessment in leukemic mice treated with MerTK inhibition. (A-B) Serum from mice treated as described in Figure 3, was subjected to Luminex for cytokines/chemokines. Analyzed using 1-way ANOVA. (NS = not significant)
